# Isolation and Molecular Profiling of Primary Mouse Retinal Ganglion Cells: Comparison of Phenotypes from Healthy and Glaucomatous Retinas

**DOI:** 10.3389/fnagi.2016.00093

**Published:** 2016-05-18

**Authors:** Sumana R. Chintalapudi, Levon Djenderedjian, Andrew B. Stiemke, Jena J. Steinle, Monica M. Jablonski, Vanessa M. Morales-Tirado

**Affiliations:** ^1^Department of Ophthalmology, The University of Tennessee Health Science CenterMemphis, TN, USA; ^2^Department of Anatomy and Cell Biology, Wayne State UniversityDetroit, MI, USA; ^3^Department of Ophthalmology, Wayne State UniversityDetroit, MI, USA; ^4^Department of Anatomy and Neurobiology, The University of Tennessee Health Science CenterMemphis, TN, USA; ^5^Department of Pharmaceutical Sciences, The University of Tennessee Health Science CenterMemphis, TN, USA; ^6^Department of Microbiology, Immunology and Biochemistry, The University of Tennessee Health Science CenterMemphis, TN, USA

**Keywords:** retinal ganglion cell, glaucoma, retinal cells, neurodegeneration, flow cytometry

## Abstract

Loss of functional retinal ganglion cells (RGC) is an element of retinal degeneration that is poorly understood. This is in part due to the lack of a reliable and validated protocol for the isolation of primary RGCs. Here we optimize a feasible, reproducible, standardized flow cytometry-based protocol for the isolation and enrichment of homogeneous RGC with the Thy1.2^hi^CD48^neg^CD15^neg^CD57^neg^ surface phenotype. A three-step validation process was performed by: (1) genomic profiling of 25-genes associated with retinal cells; (2) intracellular labeling of homogeneous sorted cells for the intracellular RGC-markers SNCG, brain-specific homeobox/POU domain protein 3A (BRN3A), TUJ1, and RNA-binding protein with multiple splicing (RBPMS); and (3) by applying the methodology on RGC from a mouse model with elevated intraocular pressure (IOP) and optic nerve damage. Use of primary RGC cultures will allow for future careful assessment of important cell specific pathways in RGC to provide mechanistic insights into the declining of visual acuity in aged populations and those suffering from retinal neurodegenerative diseases.

## Introduction

Millions of the people in the USA suffer from irreversible vision loss that is incited by retinal ganglion cell (RGC) loss due to retinal neurodegenerative diseases such as diabetic retinopathy or glaucoma and by age-related changes in neural function or behavior. A common element in the pathophysiology of both diseases and in the aging population is the loss or death of RGCs; however the cellular mechanisms underlying their loss remain unclear. This is in part due to the lack of standardized, reliable protocols to isolate large numbers of highly enriched RGCs and/or a RGC line for *in vitro* mechanistic studies (Van Bergen et al., 2009; Wood et al., 2010). Identifying the genetic basis or cellular mechanisms causing RGC degeneration would be the first step towards development of efficacious therapies to slow or reverse RGC damage, in turn preserving vision. The lack of a validated RGC population represents a large unmet need for the vision research community at large.

The isolation and enrichment of primary murine RGCs is essential for investigating RGC responses to specific therapies *in vitro*. A number of challenges have prevented progress towards the use of a homogeneous primary murine RGC population. First, a major challenge lies on the scarce number of RGCs that can be isolated from murine retinae (Dreher et al., 1985; Williams et al., 1996; Jeon et al., 1998). Second, current signature markers for the identification of RGCs are intracellular markers (Surgucheva et al., 2008; Nadal-Nicolás et al., 2009; Kwong et al., 2010; Rodriguez et al., 2014), impeding the isolation of viable, metabolically active cells downstream for *in vitro* studies. Third, current protocols are lengthy and have not been standardized for the isolation of primary murine RGCs from dissociated retinae. Barres et al. (1988) adapted the immunopanning technique into a two-step process to purify RGCs. The process includes depletion of macrophages and endothelial cells, followed by positive selection of cells responding to anti-thymocyte antigen (Thy1). Recently, Hong et al. (2012) optimized a similar process that included positive selection of Thy1^+^ cells using magnetic beads followed by cell sorting. Both approaches require lengthy isolations and their yields are inconsistent. A commercial kit is available for isolating RGCs from retinae (Pennartz et al., 2010), however, it has two major limitations. Firstly, the kit is for exclusive use in rats, yet mice are the primary animal model used in vision research. Secondly, the specificity of this kit for RGCs is debatable, as amacrine cells could also be isolated with this method. In recent years, the use of Dynabeads or flow cytometry in conjunction with monoclonal antibodies (mAbs; Jackson et al., 1990) or lectins (Sahagun et al., 1989) have provided powerful tools to improve the purity of isolated cells. Flow cytometry, also known as Fluorescence Activated Cell Sorting (FACS), is a powerful method that analyses cell suspensions and provides quantitative and qualitative data with a high level of sensitivity. FACS cellular discrimination is based on physical properties such as surface area and the internal complexity or granularity of the cells (Julius et al., 1972). Multi-dimensional analyses, based upon the expression of proteins on the cell surface as well as intracellular localization, can be performed by the combination of mAbs tagged with fluorochromes. Current FACS-based cell sorting techniques allow for the separation of up to four different cell populations based on multivariate properties. Sorted cells can be collected and are viable for downstream analyses.

In the present study, we developed a novel flow cytometry-based protocol to generate a homogeneous RGC population from murine retinae. We employed a highly stringent sort strategy coupled with qualitative PCR (qPCR) and intracellular staining with RGC-signature markers to verify the *purity and homogeneity* of the enriched population. Our isolation technique provides a powerful tool for vision research to assist in the understanding of the molecular pathways and key players in preservation of RGC function and health to develop novel therapies for vision loss.

## Materials and Methods

### Dissociation of Murine Retinae

Two hundred C57BL/6J mice between 5–7 weeks of age, 22 BXD66 mice ages 5 weeks (young) and >12 months (old) were used in this study. All procedures were approved by the Institutional Animal Care and Use Committee (IACUC) review board at the University of Tennessee Health Science Center (UTHSC) and followed the Association for Research in Vision and Ophthalmology (ARVO) Statements for the Use of Animals in Ophthalmic and Vision Research, in addition to the guidelines for laboratory animal experiments (Institute of Laboratory Animal Resources, Public Health Service Policy on Humane Care and Use of Laboratory Animals). Mice were sacrificed by cervical dislocation followed by enucleation, as previously described (Jiang et al., 2015). Retinae were dissociated using enzymatic digestion. The resultant cell suspension was filtered using a Falcon 70 μm nylon strainer (BD Biosciences, San Jose, CA, USA) followed by centrifugation at 1500 RPM × 5 min at RT. Cells were resuspended in PBS/1% FBS (Lonza, Walkersville, MD, USA) and kept on ice until ready for use.

### Flow Cytometry Analyses

#### Cell Surface Labeling

Cell viability was evaluated at the time of retinae cell dissociation. To ensure we obtain live cells after sorting, we labeled the cells with Zombie Aqua^TM^ (BioLegend, San Diego, CA, USA) a permeant dye to discriminate between live (negative for dye) and dead (positive for dye) cells. Live cells were treated with 1.0 μg of anti-CD16/CD32 per 1.0 × 10^6^ cells in 100 μL to block FcγRII/III (clone 93; BioLegend), which minimizes non-specific binding of the primary antibodies and in turn inhibits endocytosis, phagocytosis and antigen presentation due to FcγR activation. The following primary antibodies were used to detect surface antigens by incubating the cells on ice for 30 min: anti-CD90.1 PerCP-Cy5.5 (Thy1.1, clone OX-7, BioLegend, exhibits no cross-reactivity with CD90.2); anti-CD90.2 Alexa Fluor-700 (Thy1.2, clone 30-H12, BioLegend, exhibits no cross-reactivity with CD90.1); anti-CD48 PE-Cy7 (clone HM48-1, BioLegend, labels monocytes and microglia); anti-CD15 PE (clone MC-480, BioLegend, labels amacrine cells); and anti-CD57 (clone VC1.1, Sigma Aldrich, St. Louis, MO, USA also labels amacrine cells). Because the anti-CD57 antibody was unconjugated, a Brilliant violet 421-tagged secondary antibody (Life Technologies, Carlsbad, CA, USA) was used to allow for sorting.

#### Sorting Strategy

Cells were enriched by FACS using a BD Biosciences FACSAria^TM^ Cell Sorter equipped with 4-lasers (BD Biosciences, San Jose, CA, USA). In this investigation, we used the 488 nm blue, the 630 nm red and the 405 nm violet diode lasers. Dissociated cells were maintained at 4°C using a temperature controlled sample injection and collection chamber. At the time of cell sorting, we used unlabeled murine retinal cells as controls and individual samples were labeled with antibodies specific for different retinal cell surface markers. We used AbC^™^ Total Antibody Compensation Bead Kit (Life Technologies) to prepare single color controls using the manufacturer’s protocol. These are highly sensitive and efficient antibody capture beads with a broad multispecies reactivity. To determine the efficiency of the sort, we performed FACS analysis on a small aliquot from the eluted sorted cells.

#### Intracellular Labeling

Dissociated cells labeled as detailed above followed by 1 h fixation at 4°C using the BD Cytofix/Cytoperm^®^ fixation/permeabilization solution (BD Biosciences) as in Morales-Tirado et al. (2004, 2010, 2011) and Kasow et al. (2011). Cells were incubated for 1 h at 4°C in the following antibodies that were diluted in BD Perm/Wash (BD Biosciences) buffer: anti-RNA-Binding Protein With Multiple Splicing (RBPMS; rabbit polyclonal IgG; Santa Cruz Biotechnology, Santa Cruz, CA, USA; 1:100 dilution); anti-SNCG (rabbit polyclonal IgG; GeneTex, 1:100 dilution), Brain-Specific Homeobox/POU Domain Protein 3A (BRN3A; goat ployclonal IgG; Santa Cruz Biotechnology; 1:100 dilution); and anti-TUJ1 (mouse monoclonal IgG2a; Covance; 1:100 dilution). The appropriate Alexa Fluor 488 tagged secondary antibodies (1:200 dilution; Invitrogen) were used to allow for data acquisition and analysis. Cells were kept in PBS/1%FBS on ice until the time of analysis. Data acquisition was performed on a BD LSRII Flow Cytometer (BD Biosciences) and analyses were performed using FlowJo vX10.0.6 (Tree Star, Inc., Ashland, OR, USA). To confirm results we performed additional data acquisition using the Miltenyi Biotec MACSQuant^®^ 10 Analyzer (Myltenyi Biotec, San Diego, CA, USA).

#### Flow Cytometry and Confocal Microscopy Dual Analysis

Labeled retinal cells were acquired based on area and aspect ratio, gating out cell debris and cell aggregates from the analysis using the Amnis^®^ FlowSight^®^ Imager (EMD Millipore, Amnis Division, Seattle, WA, USA). Flow cytometry features include a violet (405 nm, 100 mW), blue (488 nm, 60 mW), red (642 nm, 100 mW), and side scatter (SSC; 785 nm, 8 mW) lasers. Data were analyzed in IDEAS Software after compensation of single color control samples using a compensation matrix. Confocal images were taken at 20×.

### Gene Analyses

#### RNA Isolation and cDNA Synthesis and Pre-Amplification of cDNA Template

RNA from 5.0 × 10^5^ sorted Live Thy1.2^+^ CD48^neg^CD15^neg^CD57^neg^ cells was extracted following the Qiagen^®^ miRNeasy Mini Kit (Qiagen, Valencia, CA, USA) manufacturer’s specifications. RNA concentration was assessed using a Nanodrop Spectophotometer (Nanodrop, Wilmington, DE, USA). cDNA was synthesized from RNA using the SuperScript^®^ VILO^TM^ cDNA Synthesis Kit (Life Technologies). Following the manufacturer’s instructions, we used 100 ng of RNA for each reaction. Briefly, the pre- amplification reaction mixture was prepared to discriminate among different retinal cells (Table 1) using cDNA, TaqMan^®^ PreAmp Master Mix and the pooled primer mix listed in Table 2. Pre-amplification: the enzyme activation step was carried out at 95°C for 10 min, followed by 14 cycles at 95°C for 15 s followed by 60°C for 4 min. Subsequently, the pre-amplified cDNA was diluted 1:10 in Tris EDTA buffer and was kept at −20°C until ready for use. The pre-amplification of cDNA step was crucial to increase the sensitivity of detection for downstream quantification using qPCR. As part of our stringent validation and confirmation techniques, we used a series of gene targets that are specific for different retinal cell populations, including a housekeeping gene (*Hprt*). The primers that were used for the pre-amplification step are listed in the Table 2.

**Table 1:** **Genes used in gene expression analyses as part of the validation of our RGC enrichment protocol**.

Retinal cell type	Genes expressed by retinal cell type
Retinal ganglion cell	*Pou4f1; Rbpms; Sncg; Tubb3; Chrma6; Rbfox3; Nef-H*
Amacrine	*Gad2; Fut4; Calb2; Pvalb; Slc6a9; Pcp4; Vip; Thy1*
Astrocytes	*Aqp4; Prdx6; Gfap; Slc1a3; Pax2*
Müller	*ApoE; Abca8a; Vim; Aldh1a1*
Bipolar	*Pkcα; Pcp4; Rcvrn; Slc1a2*
Horizontal	*Rcvrn; Prox1; Ntrk1; Lhx1, Lim2, Calb2*
Photoreceptors	*Nrl; Rom-1; Crx;* *Pxph2; Arr3*
(Cone and Rod)	
Retinal pigment	*Cd68; Rpe65*
Epithelial cells	
Housekeeping gene	*Hprt*

**Table 2:** **List of primers used for gene expression analyses as a component of the validation of our RGC enrichment protocol**.

Gene symbol	Taqman^®^ Gene expression assays (Primers)
*Abca8a*	Mm00462440_m1
*Aldh1al*	Mm00657317_m1
*Aqp4*	Mm00802131_m1
*Calb2*	Mm00801461_m1
*Cd68*	Mm03047340_m1
*Gad2*	Mm00484623_m1
*Hprt*	Mm01545399_m1
*Lhx1*	Mm01297482_m1
*Lim2*	Mm00624623_m1
*Nrl*	Mm00476550_m1
*Ntrk1*	Mm01219406_m1
*Pcp4*	Mm00500973_m1
*Pov4f1*	Mm02343791_m1
*Prdx6*	Mm00725435_s1
*Prkca*	Mm00440858_m1
*Prox1*	Mm00435969_m1
*Pvalb*	Mm00443100_m1
*Rbpms*	Mm02343791_m1
*Rom1*	Mm00436364_g1
*Rpe65*	Mm00504133_m1
*Slc1a3*	Mm00600697_m1
*Slc6a9*	Mm00433662_m1
*Sncg*	Mm00488345_m1
*Tubb3*	Mm00727586_s1
*Vim*	Mm01333430_m1

#### qPCR Analysis Amplification Efficiency Test

We determined the primer efficiency and amplification efficiency by the absolute quantification method using a Roche LightCycler^®^ 480 Instrument and Version 1.5.0 Software (Roche, Indianapolis, IN, USA). Pre-amplified cDNA was serially diluted to a range of concentration (1, 1:10, 1:100, 1:1000). This comparison ensured that the pre-amplification process amplified genes with a wide variation of abundance. The comparative threshold (C_T_) values were plotted against the log concentration qPCR product and the slope was calculated. The closer the slope is to −3.33, the closer the amplification efficiency is to the 100% ideal, which indicates that there is a doubling of product per cycle. In addition, we performed linear regression analysis to show the correlation between gene expression measurements from our samples for the primers we validated, and a standard curve. In this study, we used C_T_ values ranging from 15 to 30 in our assays. Three biological replicates were evaluated and the C_T_ values were normalized to the endogenous gene (*Hprt*) control and compared to the C_T_ values obtained from the pre-amplifed cDNA from murine, non-sorted, retinal cells.

#### qPCR Reaction

For qPCR reaction we prepared a final volume of 10 μL PCR reaction mixture using TaqMan^®^ Universal Master Mix, diluted pre-amplified cDNA, primers (Table 2) and Nuclease Free water. Plates were analyzed on a Roche LightCycler^®^ 480. Instrument conditions included first a hold step of 50°C for 2 min followed by 95°C for 10 min. Next, we performed 40 cycles of 95°C for 15 s followed by 60° for 1 min. All measurements were made in replicates of three. Relative quantification was performed using C_T_ after determining the values of C_T_ for the reference gene (housekeeping) and the target genes in each sample. The relative fold change (*R*_q_) was calculated using the following formula: *R*_q_ = $2_{\text{T}}^{-\Delta \text{C}}$, where ΔC_T_ = C_T_ target gene − C_T_ reference gene. Data are presented as mean ± SEM. Differences between two means were assessed with *ANOVA and Tukey’s post hoc* test (PRISM, Graph Pad, La Jolla, CA, USA). Differences were considered significant at *p* < 0.05.

### Immunohistochemistry

Murine retinal sections embedded in low melting point agarose were prepared following our published methods (Nookala et al., 2010). Briefly, tissue sections were blocked with 10% goat serum and permeabilized with 2.5% Triton X-100. The following primary antibodies were used per manufacturers conditions: RBPMS (rabbit polyclonal IgG, Santa Cruz Biotechnology, 1:100 dilution); anti- γ-synuclein (SNCG, rabbit polyclonal IgG, GeneTex, 1:100 dilution); anti-BRN3A (goat polyclonal IgG, Santa Cruz Biotechnology, 1:10 dilution); anti-Neuronal Class III β-Tubulin (TUJ1, mouse monoclonal IgG2a, Covance, Princeton, NJ; 1:100 dilution); anti-HNK-1/N-CAM (CD57, Clone VC1.1, mouse monoclonal IgM, 1:10 Dilution); and CD15 (Clone:MC-480, mouse monoclonal IgM, BioLegend, 1:25 dilution). The appropriate Alexa Fluor-tagged secondary antibodies (Invitrogen, Waltham, MA; 1:200 dilution) and TO-PRO-3 iodide (Invitrogen; 1:4000 dilution) were used to indicate the location of the antigens of interest and nuclei, respectively. Sections were viewed and images were obtained using a Nikon C1 (Nikon, NY, USA) confocal microscope within the Imaging Core Facility in the Hamilton Eye Institute. All microscope settings, including laser levels and gain, were held constant to allow for relative comparisons of signal intensity within and between experiments.

### Immunofluorescence

Live Thy1.2^+^ CD48^neg^CD15^neg^CD57^neg^ sorted cells (50,000 cells) were washed once with Hanks Balanced Salt Solution (HBSS) in followed by centrifugation at 1200 RPM × 5 min at RT. Cells were labeled with SYTO^®^59 (100 nM) for 10 min in a HBSS at 37°C followed by multiple washes with HBSS for 5 min each. Cells were plated on a glass bottom microwell dish (35 mm petri dish, 14 mm microwell; MatTek Corporation, Ashland, MA, USA) and sealed with coverslip. Analysis performed using a Nikon C1 (Nikon, NY, USA) confocal microscope within the Imaging Core Facility in the Hamilton Eye Institute.

### Optic Nerve Processing, Imaging and Counting

Eyes along with optic nerves were enucleated from mice immediately after euthanasia. Optic nerves were cut close to the globe and were fixed in 0.8% paraformaldehyde and 1.22% glutaraldehyde in 0.08 M phosphate buffer. They were subsequently rinsed in buffer and post-fixed in 1% osmium tetroxide. After dehydration, the specimens were embedded in Epon 812 plastic. Sections (0.8 μm thick) were cut on an ultramicrotome (Reichert-Jung Ultracut E Ultramicrotome), stained with p-phenylenediamine (PPD) for 30 min. Digital images were taken using 10× and 4× objectives using a MicroFire digital camera (Optronics^®^, Goleta, CA, USA) mounted onto a Nikon Eclipse E800 light microscope (Nikon). Multiple contiguous photomicrographs were taken at 60× magnification to provide a continuous representation across the optic nerve. A scale of optic nerve damage similar to that used by Clark et al. was used to assign a numeric value to the appearance of the nerve (David Cantu-Crouch, Iok-Hou Pang, Mitchell D. McCartney, Abbot F. Clark; unpublished protocol). The numeric value is based upon the presence/absence of darkly stained axoplasm and presence of gliotic scars.

### Intraocular Pressure Measurements

Intraocular pressure (IOP) was measured using the induction–impact tonometer (Tonolab tonometer, Colonial Medical Supply, Franconia, NH, USA) for rodents according to the manufacturer’s recommended procedures. When measuring IOP, the tonometer was fixed in a vertical position to a support stand by means of clamps. The mouse was gently restrained by hand on an adjustable stand, and the eye was oriented in such a way that a distance of 1–4 mm was maintained between from the tip the probe to the cornea of the eye. Six consecutive IOP readings were averaged. IOP readings obtained with Tonolab have been shown to be accurate and reproducible in various mouse strains, including DBA/2J.

## Results

### The Thy1^+^ CD48^neg^ Surface Phenotype Is not Sufficient to Identify Murine RGCs

**Figure 1:**
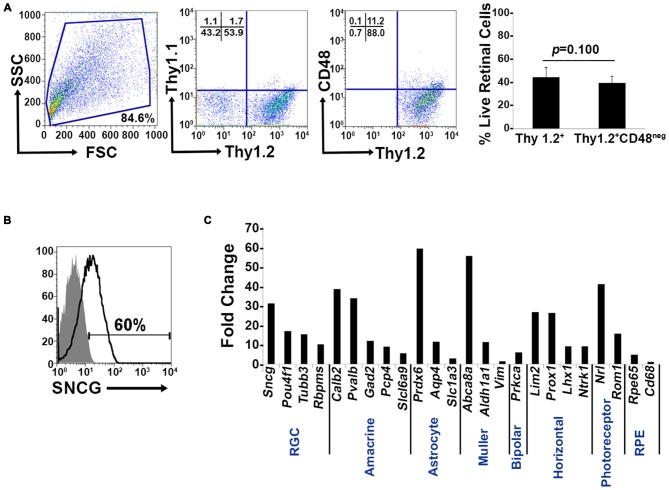
**Lack of specificity of current retinal ganglion cells (RGC) isolation methods. (A)** *Far left panel*: Pseudocolor plots were gated on live nucleated cells based on forward scatter (FSC) and side scatter (SSC) profiles. Based upon these characteristics, 84.6% of the cells were live after retinal dissociation. *Mid left panel*: A negligible number of live retinal cells were Thy1.1^+^, whereas 53.9% were Thy1.2^+^, demonstrating that in mouse Thy1.2 is the preferred surface marker. *Mid right panel*: The majority of the Thy1.2^+^ cells (88%) were CD48^neg^. *Far right panel*: There was no significant difference in the percent of live Thy1.2^+^ and Live Thy1.2^+^ CD48^neg^ cells demonstrating that the addition of CD48 as a negative selection marker is insufficient to further enrich for RGCs. **(B)** Only 60% of LiveThy1.2^+^ CD48^neg^ are SNCG^+^ indicating that many contaminating cell types remain in the LiveThy1.2^+^ CD48^neg^ cell population. Gray histogram represents control and black line represents experimental sample. **(C)** Identification of Thy1.2^+^ CD48^neg^ cells using qualitative real time PCR analysis. The expression levels of a panel of 25 genes expressed by various retinal cell types—amacrine, Müller, bipolar, horizontal, photoreceptor and retinal pigment epithelial cells—in Thy1.2^+^ CD48^neg^ cells were normalized to the levels present in unsorted total retinal cells. The Thy1.2^+^ CD48^neg^ population was contaminated with all other retinal cell types. Target gene expression levels are presented as Log_2_ fold change based on the comparative threshold (C_T_) calculation using* Hprt* as a housekeeping gene and water as negative control. Mean ± SEM; *n* = 3 biological replicates were performed in triplicate.

Murine retinal cells express two distinct isoforms of Thy1—Thy1.1 and Thy1.2 (Reif and Allen, 1964; Watanabe et al., 1981; Haeryfar and Hoskin, 2004). We compared the binding of Thy1.1 and Thy1.2 to dissociated murine retinal cells and show the majority of murine retinal cells exhibit immunoreactivity against Thy1.2 but not Thy1.1 (Figure 1A, Thy1.2^+^ at 53.9% vs. Thy1.1^+^ at 1.7%). Current RGC isolation techniques use selection based on Thy1-positivity and CD48-negativity by antibody-capture (Barres et al., 1988; Hong et al., 2012) or magnetic cell isolation (Sahagun et al., 1989; Pennartz et al., 2010). Our data demonstrate that a small percent of Thy1.2^+^ cells were also immunopositive for CD48 (11.2%) and that inclusion of CD48 as a negative selection marker did not significantly decrease the number of Thy1.2^+^ cells (Figure 1A, *right panel*). To determine if the Thy1.2^+^ CD48^neg^ cells were RGCs, we probed the cells for the expression of the RGC signature marker γ-synuclein (SNCG). Consistently, we found that a large proportion of Thy1.2^+^ CD48^neg^ cells were not SNCG^+^. Figure 1B illustrates that 60% of Live Thy1.2^+^ CD48^neg^ cells were positive for SNCG, suggesting that there is ~40% contamination by non-SNCG^+^ cell types. To address which non-RGC cell types are present within the Thy1.2^+^ CD48^neg^SNCG^neg^ phenotype, we performed qPCR analysis on the sorted cells. Results shown in Figure 1C reveal the degree of heterogeneity of the enriched RGCs using only Thy1.2 and CD48 as surface markers. Specifically, Thy1.2^+^ CD48^neg^ cells expressed genes associated with multiple retinal cells, including amacrine, Müller, bipolar, horizontal, photoreceptors and retinal pigment epithelial cells. Table 1 lists the specific genes associated with the various cell types. Our data demonstrates that selection based solely on Thy1 and CD48 expression is insufficient to isolate highly enriched RGCs.

### Confirmation of Additional Surface Markers to be Used as Negative Selectors and Intracellular RGC Markers

**Figure 2:**
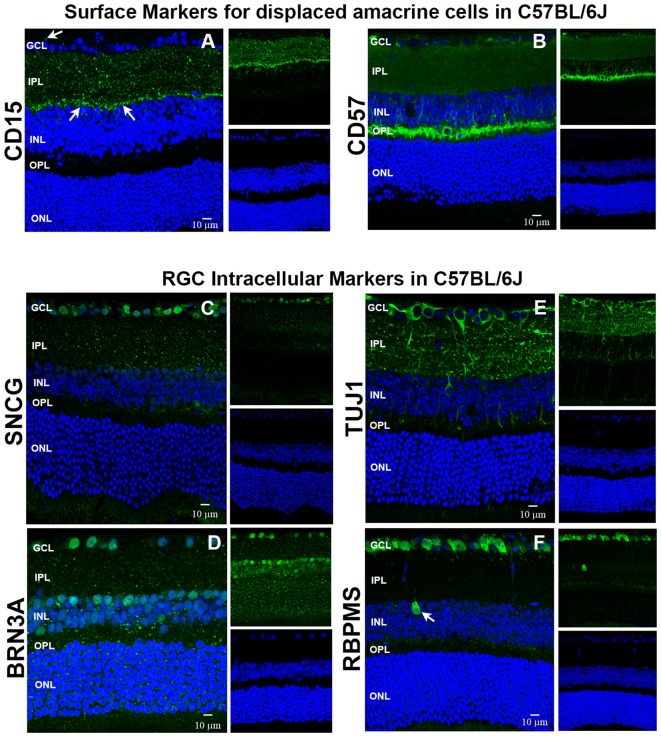
**Immunohistochemical localization of surface antigens and RGC markers used in our RGC sorting protocol.** Cellular localization of the surface proteins CD15 and CD57, as well as the intracellular proteins SNCG, BRN3A, TUJ1 and RBPMS in retinae from C57BL/6J mice. Sections from C57BL/6J mouse retinae were labeled with antibodies against **(A)** CD15, **(B)** CD57, **(C)** SNCG, **(D)** BRN3A, **(E)** TUJ1, and **(F)** RBPMS. TO-PRO-3 iodide staining labeled nuclei of all retinal cells (blue). Abbreviations: GCL, ganglion cell layer; IPL, inner plexiform layer; INL, inner nuclear layer; OPL, outer plexiform layer; ONL, outer nuclear layer. Scale bar: 10 μm.

We used immunohistochemistry (IHC) to determine the retinal localization patterns of other surface markers that were expressed by contaminating cells. CD15 has been described as a marker of retinal interneurons including amacrine and bipolar cells (Jakobs et al., 2003), while CD57 has been shown to label glial cells and photoreceptors (Uusitalo et al., 2003). Our data demonstrate that CD15 (Figure 2A) is localized in the interface between the inner nuclear layer (INL) and inner plexiform layer (IPL) and in the proximal INL (arrows), where amacrine cells are located. Occasional CD15^+^ cells were observed in the ganglion cell layer (GCL), which are most likely displaced amacrine cells (arrows). CD57 immunoreactivity (Figure 2B) was abundant in the outer plexiform layer (OPL) and in a radial pattern through the INL. Punctate staining was also present in the GCL, which are likely astrocytes or displaced amacrine cells. Collectively, these results show that these surface markers—CD15 and CD57—can be used as negative selectors to remove non-RGCs from the Thy1.2^+^ CD48^neg^ cell population.

To verify that the commonly accepted RGC intracellular markers are specific for RGCs (Jackson et al., 1990; Surgucheva et al., 2008; Nadal-Nicolás et al., 2009; Kwong et al., 2010), we labeled murine retinal sections with antibodies against SNCG, BRN3A (*Pouf4l*), TUJ1 [neuron-specific class III beta tubulin (*Tubb3*)] and RBPMS. SNCG showed abundant expression in the GCL (Figure 2C). Abundant BRN3A (Figure 2D) labeling was observed in the GCL. However, multiple cells in the INL that border the IPL are also immunopositive for BRN3A. Based upon their location, they are likely amacrine cells. TUJ1 (Figure 2E) was very abundant in the GCL and in radial labeling patterns throughout the retina that extend up to the ONL. Lastly, RBPMS was highly expressed by cells in the GCL (Figure 2F) and a small subpopulation of cells in the INL (arrow). Collectively, these data demonstrate that SNCG-, BRN3A-, TUJ1- and RBPMS-positive cells are present in the GCL and can be used to validate the identity of the cells that we isolate using our array of cell surface markers. Even though BRN3A and RBPMS also label a small subset of (likely) amacrine cells, we are confident that requiring all enriched RGCs to express all four RGC markers will yield a nearly pure RGC population.

### Live Thy1.2^+^ CD48^neg^CD15^neg^CD57^neg^ RGC Express All Signature Intracellular Markers SNGC, BRN3A, TUJ1 and RBPMS

**Figure 3:**
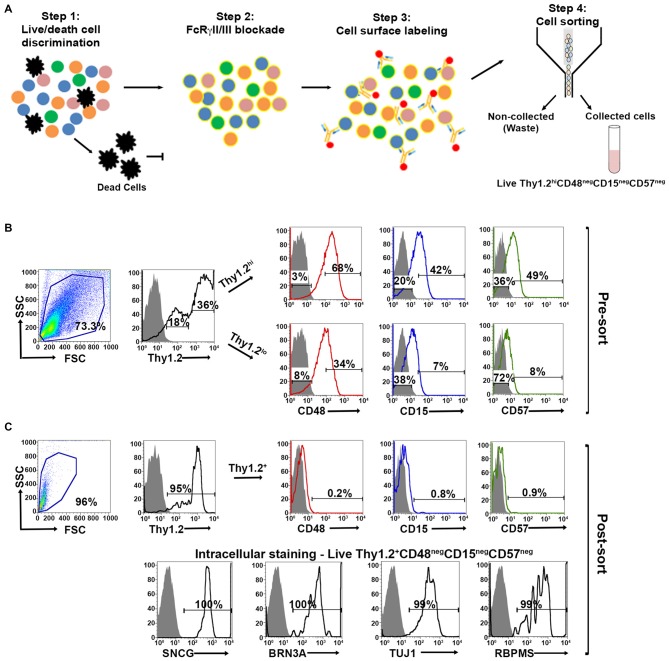
**Optimized fluorescent activated cell sorting (FACS)-based cell sorting strategy. (A)** Schematic representation of RGC isolation by flow cytometry using multiple surface markers. Step 1: Cells were labeled with Zombie Aqua^TM^ for live/dead cell analysis followed by step 2: blocking of FcγRII/III (mouse anti-CD16/32) to minimize non-specific labeling. Step 3: Cell surface labeling was performed using the following antibody cocktail: anti-mouse anti-CD90.2 Alexa Fluor 700; anti-CD48 PE-Cy7; anti-CD15 PE and anti-CD57 Brilliant Violet 421 to yield LiveThy1.2^+^ CD48^neg^CD15^neg^CD57^neg^ cells. Single labeled fluorochrome-beads were used as controls. **(B)** Surface marker expression of pre-sorted retinal cells. *Far left panel*: Live retinal cells show two distinct populations of Thy1.2 (Thy1.2^hi^ and Thy1.2^low^) based upon expression levels per cell. *Right panels*: Representative FACS plots show the expression of surface markers used for negative selection—CD48, CD15, CD57—in pre-sorted Thy1.2^hi^ and Thy1.2^low^ retinal cells. Labeled retinal cells were sorted for Thy1.2^hi^CD48^neg^CD15^neg^CD57^neg^ population. Selection for sorting included the positive selection of Thy1.2^hi^ (36%). This population was further selected for CD48^neg^ (3%), followed by CD15^neg^ (20%) and CD57^neg^ (36%). **(C)** Purity of the sorted RGCs based on the surface marker and intracellular RGC markers. *Far left panel*: LiveThy1.2^+^ CD48^neg^CD15^neg^CD57^neg^ cells show expression of Thy1.2^+^ (95%). Top right panels present representative FACS plots show the negligible expression of surface markers in sorted LiveThy1.2^+^ CD48^neg^CD15^neg^CD57^neg^ cells, demonstrating the efficiency of the sort. *Lower row* panels show representative FACS plots of the expression of intracellular RGC markers in LiveThy1.2^+^ CD48^neg^CD15^neg^CD57^neg^ cells. Ninety nine to hundred precentage of all cells express SNCG, BRN3A, TUJ1 and RBPMS, all well characterized RGC markers. The sum of gated and non-gated cells in each histogram totals 100%. This new sorting method (Thy1.2^hi^CD48^neg^CD15^neg^CD57^neg^) shows improvement over the previously used methodology (LiveThy1.2^+^ CD48^neg^) using additional surface markers. Grey indicates isotype controls, colored solid lines indicates experimental samples.

Our expanded cell sorting strategy is presented in Figure 3A. Because Thy1.2^+^ CD48^neg^ cells expressed many markers associated with retinal cells other than RGCs, we added additional surface markers—CD15 and CD57—to our sorting strategy to remove these contaminating cells. Our methodology included the negative selection of these cell surface markers to enrich for naïve RGCs that could be used in downstream analyses. Collectively, we were targeting the removal of monocytes, as well as glial, amacrine and photoreceptor cells. Prior to cell surface labeling, we added purified mouse anti-CD16/32 antibody to block FcγRII/III, thus reducing false positive immunoreactivity (Unkeless et al., 1979; Balogh et al., 2002). Our flow cytometry-based cell sorting validation studies included examination of pre- (Figure 3B) and post-sorted cells (Figure 3C) to confirm that the post-sorted cells that were isolated using cell surface markers expressed all four RGC intracellular markers: SNCG, BRN3A, TUJ1, and RBPMS. Consistently, we observed 99–100% positivity for all of the intracellular markers in the post-sorted cells, demonstrating the isolated cells were very highly enriched, if not pure RGCs. We ensured consistency of our results across multiple systems using two other cytometer systems, the MACSQuant^®^ Analyzer 10 and the FlowSight^®^. Because our results were reproducible across three different flow platforms, we are confident that our novel method is standardized (Supplementary Figures S1 and S2).

Using confocal microscopy, we investigated the morphological appearance of the sorted cells. Live Thy1.2^+^ CD48^neg^CD15^neg^CD57^neg^ cells were stained with SYTO^®^ 59 dye showing nuclear integrity. Cells showed bright blue fluorescence and strong intracellular staining (Supplementary Figure S3). This result confirms Live Thy1.2^+^ CD48^neg^CD15^neg^CD57^neg^ sorted cells show the morphology associated with RGC.

### Validation of the Enriched RGC Population by qPCR Analyses

**Figure 4:**
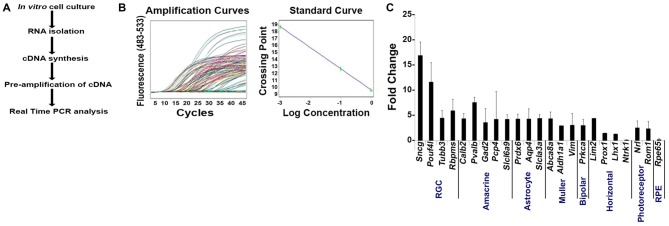
**Validation of our improved protocol for the enrichment of RGCs. (A)** Schematic representation of our validation analyses of Live Thy1.2^+^ CD48^neg^CD15^neg^CD57^neg^ sorted cells. **(B)** Amplification and standard curves to evaluate primer efficiency. *Left panel*: amplification curves of the 10-fold dilution series for all the primers. *Right panel*: Standard curves depicting C_T_ plotted against the log of the starting quantity of template for each dilution. The efficiency of the all PCR reactions was between 90–100% (−3.6 ≥ slope ≥ −3.3). All samples were prepared and analyzed in triplicate. **(C)** Validation of Live Thy1.2^+^ CD48^neg^CD15^neg^CD57^neg^ cells using qPCR analyses. Graph depicts expression of different retinal cells markers in Live Thy1.2^+^ CD48^neg^CD15^neg^CD57^neg^ that were normalized to the mRNA expression measured in total primary murine retinal cells. Genes associated with RGCs—*Sncg, Pouf41, Tubb3 and Rbpms*—showed increased gene expression compared to non-RGC associated genes. Target gene expression levels are presented as Log_2_ fold change based on C_T_ calculation using *Hprt* as housekeeping gene and water as negative control. Mean ± SEM; *n* = 3 biological replicates were performed in triplicate.

To determine if the enriched RGC population isolated using our improved strategy contained contaminants from other retinal cell populations, we measured mRNA levels by qPCR analysis using the same primers that we used on Thy1.2^+^ CD48^neg^ cells (Figure 1C). Our method is presented in Figure 4A. Using the absolute quantification method, we determined the optimal concentration of pre-amplified samples that would be suitable for our validation step and calculated the efficiency of amplification (Figure 4B). qPCR analyses (Figure 4C) shows that the highly enriched RGC population had a many fold increase in the expression of all four RGC intracellular markers: *Sncg*, *Pouf4l*, *Tubb3* and *Rbpms*. As expected, genes found in other retinal cells were expressed at significantly lower levels than in unsorted retinal cells. Moreover, our improved sorting methodology removed the contaminating retinal cell types that were present in the Thy1.2^+^ CD48^neg^ cells (compare Figures 1C, 4C). These mRNA analyses further validated our RGC enrichment strategy.

### Highly Enriched RGCs can be Isolated from A Mouse Model with Elevated IOP and Optic Nerve Damage

**Figure 5:**
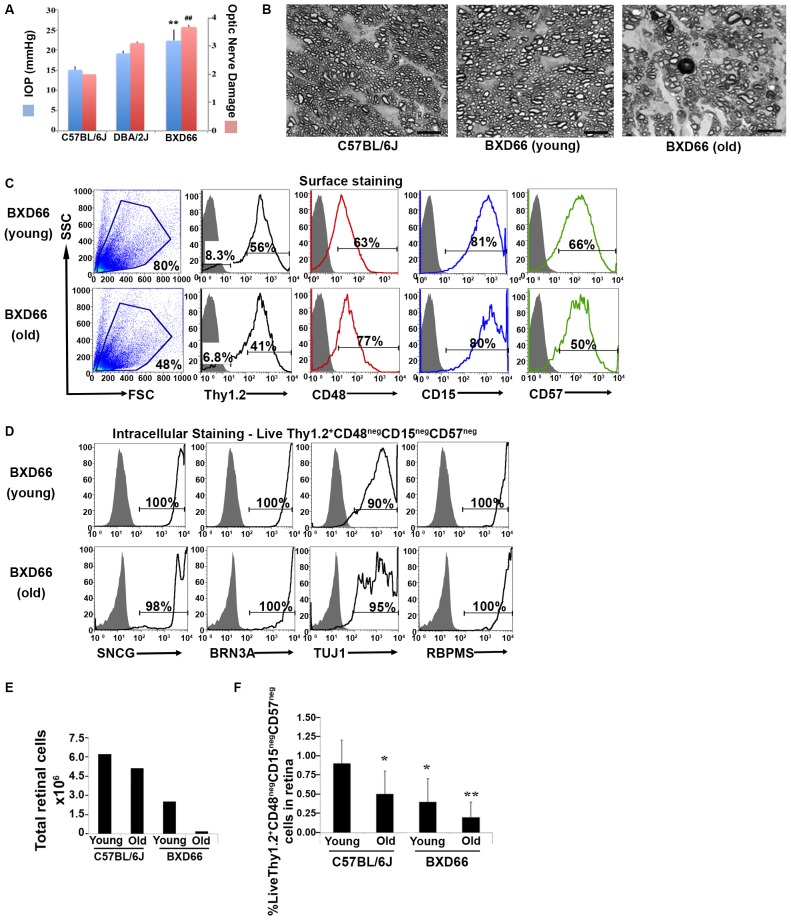
**Fewer RGCs are harvested from mice with documented elevated intraocular pressure (IOP) and RGC damage. (A)** Peak IOP and optic nerve damage distributions for C57BL/6J, DBA/2J and BXD66 mice. BXD66 mice had significantly higher peak IOP (blue bars) and optic nerve damage (pink bars) compared to C57BL/6J mice (**p < 0.005 between B6 and BXD66 IOP values; ^##^* p* < 0.005 between B6 and BXD66 optic nerve damage grade). Mean ± SEM; *n* = 4 per group. **(B)** Representative p-phenylenediamine (PPD)-stained optic nerve cross-sections from C57BL/6J (young: 5–7 weeks old), BXD66 (young: 5 weeks old), and BXD66 (>12 months old) mice. Both young C57BL/6J and BXD66 mice had a low degree of damage. In contrast, optic nerves from old BXD66 mice presented with disorganized axon bundles, increased glial scarring and multiple dead/dying axons. Scale bar = 10 μm. **(C)** Characterization of enriched RGCs from young and old BXD66 mice obtained through our improved flow sorting method. Representative histograms show comparison of surface expression of Thy1.2, CD48, CD15 and CD57 in total retinal cells of young vs. old BXD66 mice. *Top panel*: In BXD66 young mice, live retinal cells have the following abundance levels: Thy1.2 (56%), CD48 (63%), CD15 (81%) and CD57 (66%). *Bottom panel*: In BXD66 old mice, live retinal cells have the following abundance levels: Thy1.2 (41%), CD48 (77%), CD15 (80%) and CD57 (50%). Gray histogram indicates isotype controls, black solid lines indicates antibody labeling. **(D)** Relative purity of sorted RGCs based upon surface marker selection and intracellular RGC markers. Representative histograms show comparison of intracellular expression of RGC markers SNCG, BRN3A, TUJ1 and RBPMS in Live Thy1.2^+^ CD48^neg^CD15^neg^CD57^neg^ cells of young and old BXD66 mice. Top panel: In BXD66 young mice have the following abundance levels: SNCG (100%), BRN3A (100%), TUJ1 (90%) and RBPMS (100%). Bottom panel: In BXD66 old mice have the following abundance levels: SNCG (98%), BRN3A (100%), TUJ1 (95%) and RBPMS (100%). Gray histogram indicates isotype controls, black solid lines indicates antibody labeling. **(E)** Number of cells per retina obtained from dissociated retinae from young and old mice. **(F)** Percentage of Live Thy1.2^+^ CD48^neg^CD15^neg^CD57^neg^ cells in retinae from C57BL/6J (young and old) and BXD66 (young and old) mice. **p < 0.005; *p < 0.05 compared to young C57BL/6J mice. Mean ± SEM; *n* = 3 per group.

We sought to apply our improved RGC isolation methodology on a retinal degenerative disease model. Members of our research group have analyzed a family of over 100 BXD (Peirce et al., 2004) murine strains and their parental strains—C57BL/6J and DBA/2J—at five different age cohorts to determine which strains had the phenotype of optic nerve damage and elevated IOP. Both of these phenotypes are associated with retinal degeneration in glaucoma. Our examination revealed that the BXD66 strain had both elevated IOP and higher optic nerve damage compared to both C57BL/6J and DBA/2J parental strains of mice (Figure 5A). In addition, the optic nerves of old BXD66 mice had an increase in axon damage and glial scarring compared to young C57L/6J and BXD66 mice. Figure 5B depicts representative micrographs showing optic nerve damage.

To determine if our RGC protocol was valid using retinae with damaged RGCs, we applied our optimized and validated enrichment strategy on retinae obtained from young and old BXD66 mice. We compared two different ages to investigate if our enrichment protocol could be used on mice with compromised RGCs and if the efficiency of the strategy was age dependent. We first compared the cellularity of retinal cells and the percentage of live cells between the two ages of mice. We consistently found a marked reduction in the number of live retinal cells between BXD66 old (>12 months old) and young (5 weeks old) mice (Figure 5E; live cells young vs. old: 80% vs. 48%). It is worth noting that the percentage of live cells isolated from young pre-degenerative BXD66 mice (5 weeks old) is comparable to that obtained from young C57BL/6J (5–7 weeks old) mice (compare to Figure 1A). Selecting for the phenotype Live Thy1.2^+^ CD48^neg^CD15^neg^CD57^neg^, enriched RGCs (Figure 5C) were examined for the presence of the same signature intracellular RGC markers that we used throughout this investigation. Similar to that found in C57BL/6J mice, the majority of the enriched cells from BXD66 mice are immunoreactive toward the four intracellular RGC markers (Figure 5D). However, we observed a slight reduction in TUJ1 in BXD66 young and old mice compared to the C57BL/6J parental line, which may be indicative of RGC damage in this model. When comparing the total number of retinal cells (obtained from retinae of young and old C57BL/6J and BXD66 mice) with those of the phenotype Live Thy1.2^+^ CD48^neg^CD15^neg^CD57^neg^, we found similar patterns of changes (compare both panels in Figure 5F). Within a strain, retinae from old mice had fewer live retinal cells than young mice. In addition, retinae from BXD66 mice had a reduced number of retinal cells compared to age-matched C57BL/6J mice (Figure 5F). Moreover, old C57BL/6J mice had a lower percentage of Thy1.2^hi^CD48^neg^CD15^neg^CD57^neg^ retinal cells compared to young mice (0.9% ± 0.3 in young mice compared to 0.5% ± 0.3 in old mice, *p* = 0.041, Figure 5F). Retinae from young BXD66 mice also had a higher, but not significantly different, percentage of Thy1.2^hi^CD48^neg^CD15^neg^CD57^neg^ cells compared to old BXD66 mice (0.4% ± 0.3 in young mice compared to 0.2% ± 0.2 in old mice, *p* = 0.177). Lastly, there was a significant reduction in the percentage of Live Thy1.2^hi^CD48^neg^CD15^neg^CD57^neg^ cells in retinae from young BXD66 mice compared to young C57BL/6J (0.9% ± 0.3 in C57BL/6J mice compared to 0.4% ± 0.3 in BXD66 mice, *p* = 0.024). Collectively, our data show that our improved RGC enrichment protocol is also effective on retinae from old mice with no retinal degeneration (C57BL/6J) and in mice with a phenotype of elevated IOP and RGC damage (BXD66).

## Discussion

Evidence suggests the vulnerability of the ganglion cells to aging (Jackson and Owsley, 2003; Calkins, 2013). Age-related degeneration contributes to decrease in visual function, contributing to impairment and reduction in health-related quality of life. The physiological function of the ganglion cells in aging is affected by a myriad of factors including changes in morphology, anatomy and at the subcellular level (Spear, 1993; Samuel et al., 2011; Calkins, 2013). Similarly, several retinal neurodegenerative diseases have the common factor of loss of ganglion cells and their axons (Garcia-Valenzuela et al., 1995; Weber et al., 1998; Osborne et al., 1999; Morgan et al., 2000; van Dijk et al., 2009, 2010). The understanding of the mechanisms underlying these processes is limited by the lack of a simple *in vitro* system with which to study RGC function. This is in part is due to the scarce number of RGCs and the heterogeneity present among the current cellular enrichment protocols. The use of antibody-dependent plate adhesion, or immunopanning, originated more than 35 years ago when it was used for the enrichment of immune cells. Barres et al. (1988) utilized the immunopanning system wherein they used Thy1 and CD48 to isolate RGCs. They demonstrate the following three points: (1) Thy1 is not an exclusive marker of murine or rat RGCs, as other Thy1^+^ retinal cells do not show the morphology and/or electrophysiological characteristics of RGCs; (2) there is variation in the expression of Thy1 positivity among RGCs; and (3) this assay only produced a 95% efficiency based on Thy1 expression. This classical study allowed for the first use of immune techniques in the field of neuroscience. Prior to the publishing of this methodology, RGC isolation was based on density gradient centrifugation with varying yield degrees (Kornguth et al., 1981; Beale et al., 1983; Sarthy et al., 1983). Although inexpensive and rapid, a major drawback of Barres’s methodology was that it required the retrograde labeling of fast blue into both superior collicular and brachia 48 h prior to retinal dissection. Shoge et al. (1999) developed a protocol for the enrichment of rat RGCs based on magnetic cell separation via inclusion of Thy1^+^ cells and the exclusion of macrophages. This method became popular because it provided a fast method for enrichment of rat RGCs, although it required magnetic columns and magnetic drivers for the separation. Unfortunately, their RGC enrichment was only 31% indicating that many contaminating cells are present. Recently, Hong et al. (2012) combined the immunopanning and magnetic cell sorter techniques to isolate murine postnatal (P1–4) RGCs. However, because the success of the method was based solely on exclusion of glial fibrillary acidic protein and synthaxin 1, the purity of the RGCs that were isolated is unknown.

We sought to develop an improved RGC enrichment strategy that could be used by most investigators without the requirement for retrograde labeling that may alter the physiology of RGCs. Our protocol also does not include immunopanning which is lengthy. Our method is rapid and requires only 5 h from the initiation of retinal dissection to the completion of fluorescence activated cell sorting. Because of the exquisite sensitivity of the method, FACS-based sorting is suited for purification of cells that comprise a very small percentage within a cell suspension. Thus, the procedure is ideal for the isolation and enrichment of RGCs, estimated to be about 0.5% of retinal cells (Dreher et al., 1985; Jeon et al., 1998). A caveat of our methodology is the requirement of expensive FACS instrumentation, and the need for highly trained and specialized operator. However, most academic and research facilities have flow cytometry core facilities, which should make this method accessible to most investigators. FACS-based sorting has a tremendous versatility because of the large number of selection markers that can be used in the enrichment process. Typically, the selection is based on 2–15 complementary parameters, which allow for acquisition of a reproducible and homogeneous phenotype within a sample and between samples that can be used in follow up *in vitro* studies. This methodology also allows for the immediate identification of viable cells and excludes cells that are undergoing apoptosis or are already dead. In addition, the inclusion of an Fc receptor blocker allows for exclusion of microglia, monocytes and macrophages, which results in a higher degree of RGCs. Lastly, another advantage of this methodology is the immediate verification of the sort purity, as it only takes as small aliquot of the isolated product to verify the surface phenotype.

As part of our RGC enrichment strategy, we incorporated robust validation components. For example, within our validation studies, we use IHC to validate two additional surface markers—CD15 and CD57—that improved the efficiency of our sorting strategy. In addition, the stringency of FACS validation strategy was confirmed using four RGC specific intracellular markers—*Sncg*, *Pouf4l*, *Tubb3*, and *Rbpms*, which encode for SNCG, BRN3A, TUJ1, and RBPMS, respectively—both at the protein and mRNA level. Furthermore, we identified the presence of non-RGCs using multiple genes expressed by other retinal cell types using qPCR analyses. To our knowledge, this is the first time, such a stringent validation process using both gene expression (qPCR) and protein (flow cytometry and IHC) analyses have been performed on isolated and enriched murine RGCs.

As a further validation of the applicability of our enrichment protocol, we evaluated the ability of our RGC enrichment strategy to isolate RGCs from a mouse model with elevated IOP and optic nerve damage. Members of our collaborative group use the BXD family of recombinant inbred (RI) mice for gene mapping and quantitative trait locus (QTL) analyses to identify specific genomic regions that modulate various glaucoma-associated endophenotypes (Lu et al., 2011; Swaminathan et al., 2013; Templeton et al., 2013). The BXD RI strains are derived by inbreeding the C57BL/6J and DBA/2J parental strains. BXD mice have been successfully used in vision research to elucidate specific cause-effect predictions between genes and a quantitative phenotype, such as differences in expression levels (Peirce et al., 2004; Dong et al., 2007; Lu et al., 2008). In these studies, we selected the BXD66 strain due to its age-dependent elevation in IOP (Figure 5A) and optic nerve damage (Figure 5B). Our intracellular flow cytometry protein analysis revealed a similar percentage of cells that show positivity for the surface marker Thy1.2, while negative selection of CD48, CD15 and CD57, and concomitant positivity for the intracellular markers—SNCG, BRN3A, TUJ1, and RBPMS—in both C57BL/6J mice and BXD66, irrespective of the age bin (5 weeks vs. 12 months). Nonetheless, the percentage of RGCs was significantly lower in the BXD66 mice compared to the C57BL/6J mice, suggesting that RGCs, a population that is already scarce in the mammalian retina, is less abundant in BXD66 mice. Our data also highlight variation in the expression of TUJ1 in RGCs isolated from BXD66 mice at different ages, suggesting that differences in TUJ1 levels likely reflect altered physiology of the damaged RGCs, rather than the absence of RGCs in these mice.

In summary, we demonstrate a powerful technique for the isolation and enrichment of primary murine RGC with the phenotype Live Thy1.2^hi^CD48^neg^CD15^neg^CD57^neg^, which concomitantly express the RGC signature intracellular markers SNCG, RBPMS, TUJ1, and BRN3A. These cells can be used for controlled *in vitro* studies of RGCs derived from healthy and disease models. The streamlined and effective isolation and validation method described here will facilitate subsequent research on the pathological and pharmacological processes with clinical relevance to diseases involving RGCs.

## Author Contributions

SRC conducted experiments, participated in data interpretation, discussion, drafted manuscript, and approved final version to be published. LD, conducted experiments, participated in data interpretation, drafted manuscript, and approved final version to be published. ABS conducted experiments, analyze and interpret work, and approved final version to be published. JJS participated in data analysis, interpretation, draft and final approval of the manuscript. MMJ participated in the conceptualization of the project, participated in data interpretation, drafted manuscript, approved final version to be published, and is accountable for all aspects of the work. VMM-T participated in the conceptualization of the project, conducted and supervised experiments, participated in data analysis and interpretation, drafted manuscript, approved final version to be published, and is accountable for all aspects of the work.

## Funding

This study was funded by the following: Juvenile Diabetes Research Foundation, Priority Grant (VMM-T, JJS); National Eye Institute EY021200 (MMJ); Gerwin Fellowship (VMM-T); Department of Defense US Army Medical Research and Materiel Command (VMM-T, JJS); Research to Prevent Blindness (PI: JCF).

## Conflict of Interest Statement

The authors declare that the research was conducted in the absence of any commercial or financial relationships that could be construed as a potential conflict of interest.

## Supplementary material

Supplementary Figure 1 **Consistency of flow cytometry results.** Dissociated murine retinal cells from C57BL/6J mice were labeled for cell surface markers as in Figure 3. In contrast to the data presented in Figures 1, 3, 5, this sample was acquired using a MACSQuant^®^ Analyzer 10 from Miltenyi Biotec. Results show consistency between the two different cytometers.

Supplementary Figure 2 **Intracellular expression of the RGC marker RBPMS in Live Thy1.2^+^ CD48^neg^CD15^neg^CD57^neg^ cells.** Representative images from a cohort of 250,000 pictures at 20× of cells using the FlowSight^®^ Imager. Results show the localization of Thy1.2 surface and RBPMS intracellular markers in Live Thy1.2^+^ CD48^neg^CD15^neg^CD57^neg^ cells.

Supplementary Figure 3 **Live Thy1.2^+^ CD48^neg^CD15^neg^CD57^neg^ show RGC morphology. (A)** Representative image of sorted cells after being maintained overnight at 37°C/5%CO_2_ in RGC culture media. **(B)** Representative images of sorted cells immediately after sort.
