# Supplementary figures and images for: Isolation and Molecular Profiling of Primary Mouse Retinal Ganglion Cells: Comparison of Phenotypes from Healthy and Glaucomatous Retinas
Source: Front Aging Neurosci. 2016 May 18;8:93. doi: 10.3389/fnagi.2016.00093 (PMC4870266; doi:10.3389/fnagi.2016.00093)

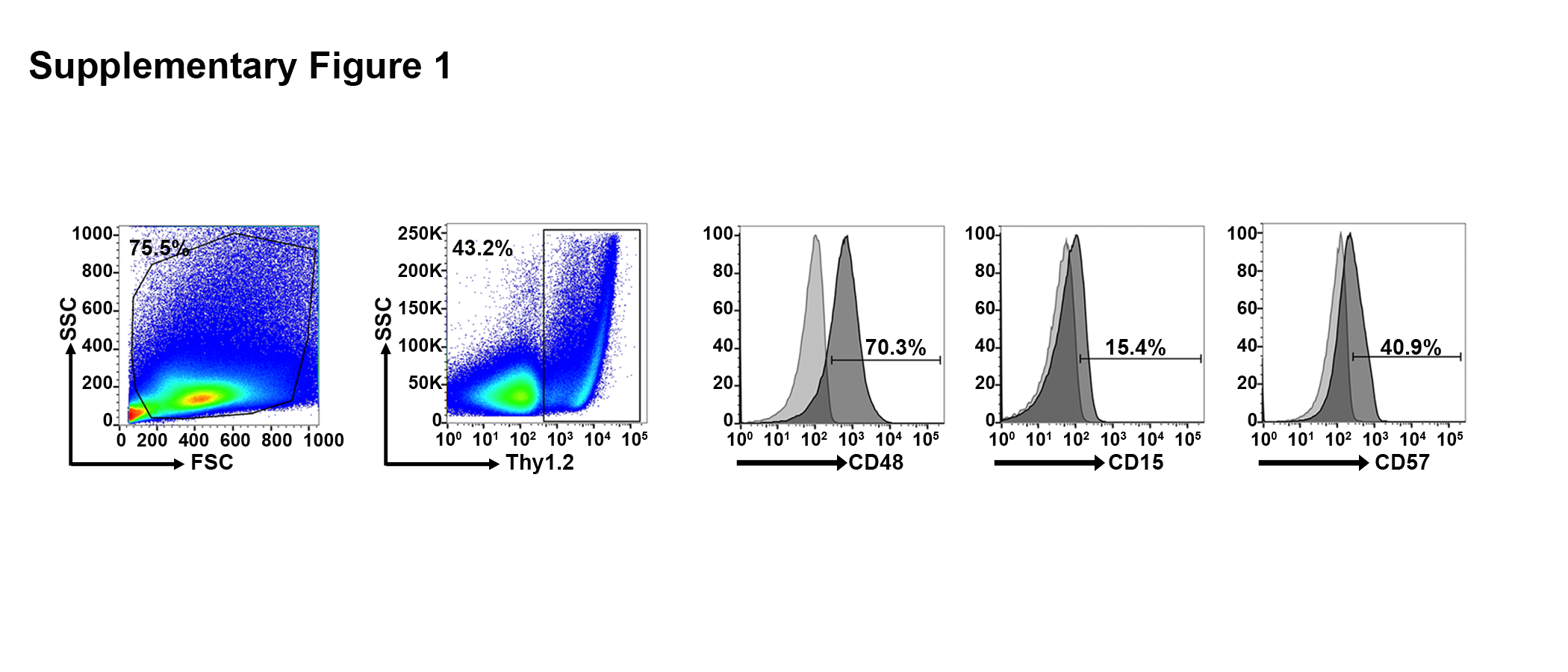

Supplement: Supplementary Figure 1 — Consistency of flow cytometry results. Dissociated murine retinal cells from C57BL/6J mice were labeled for cell surface markers as in Figure 3. In contrast to the data presented in Figures 1, 3, 5, this sample was acquired using a MACSQuant® Analyzer 10 from Miltenyi Biotec. Results show consistency between the two different cytometers. [file Image_1.tif]

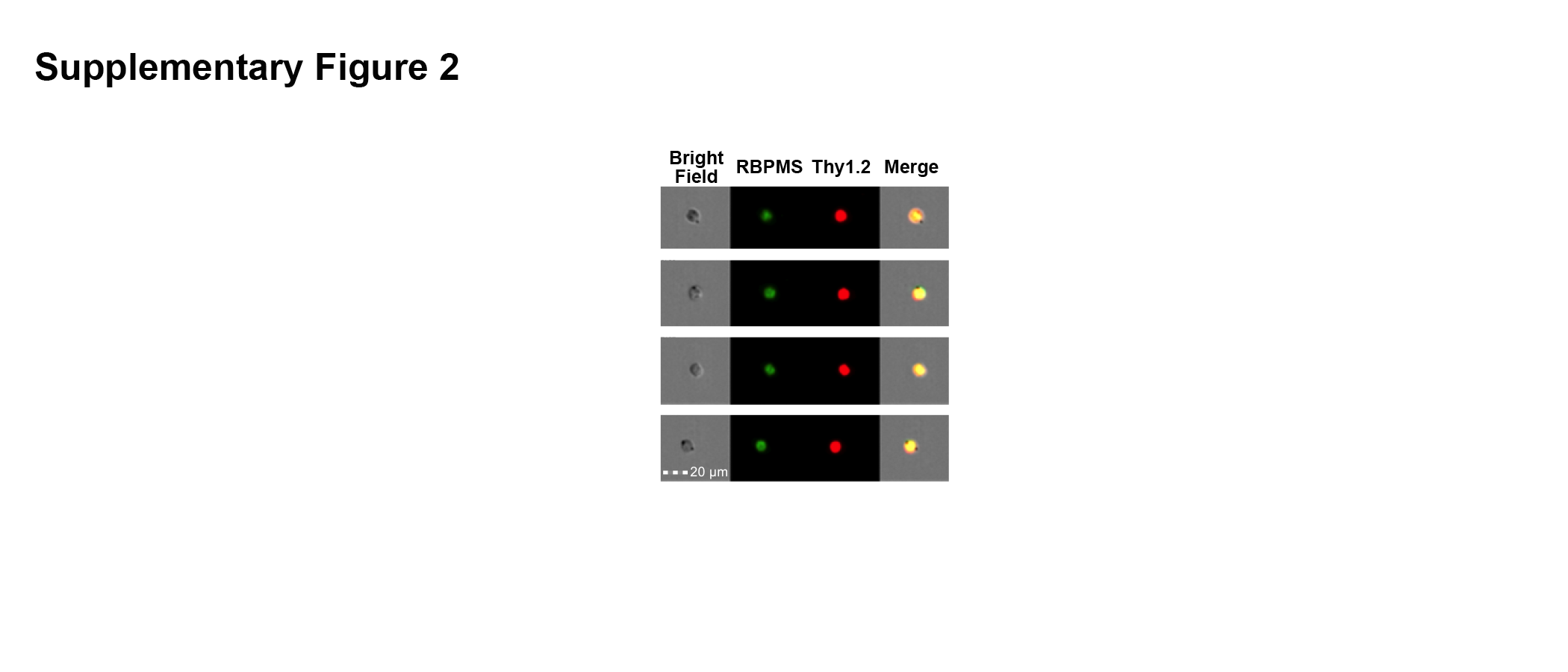

Supplement: Supplementary Figure 2 — Intracellular expression of the RGC marker RBPMS in Live Thy1.2+ CD48negCD15negCD57neg cells. Representative images from a cohort of 250,000 pictures at 20× of cells using the FlowSight® Imager. Results show the localization of Thy1.2 surface and RBPMS intracellular markers in Live Thy1.2+ CD48negCD15negCD57neg cells. [file Image_2.tif]

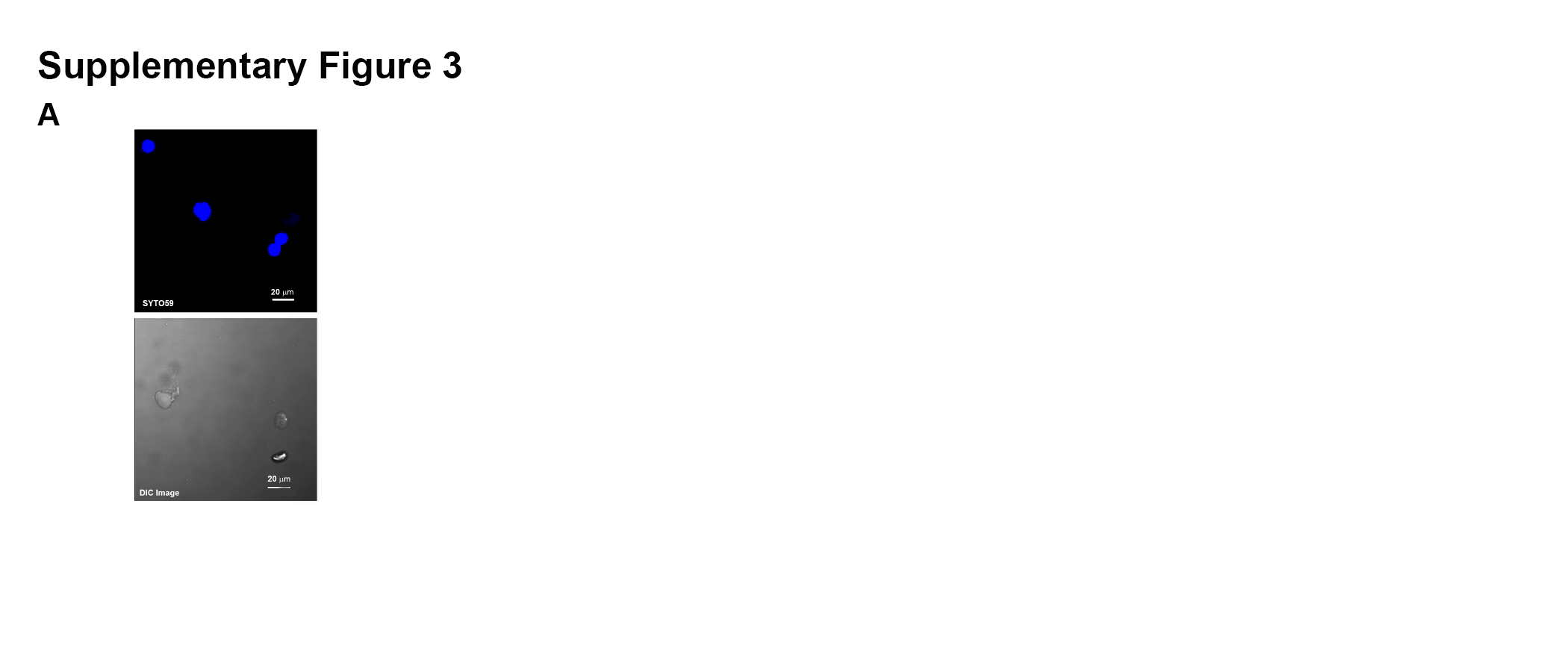

Supplement: Supplementary Figure 3 — Live Thy1.2+ CD48negCD15negCD57neg show RGC morphology. (A) Representative image of sorted cells after being maintained overnight at 37°C/5%CO2 in RGC culture media. (B) Representative images of sorted cells immediately after sort. [file Image_3.tif]
